# Effectiveness of BNT162b2 BA.4/5 Bivalent COVID-19 Vaccine against Long COVID Symptoms: A US Nationwide Study

**DOI:** 10.3390/vaccines12020183

**Published:** 2024-02-11

**Authors:** Manuela Di Fusco, Xiaowu Sun, Kristen E. Allen, Alon Yehoshua, Alexandra Berk, Mary B. Alvarez, Thomas M. Porter, Jinma Ren, Laura Puzniak, Santiago M. C. Lopez, Joseph C. Cappelleri

**Affiliations:** 1 Pfizer Inc., New York, NY 10001, USA; 2 CVS Health, Woonsocket, RI 02895, USA

**Keywords:** BNT162b2, bivalent, BA.4/5, long COVID, PASC, COVID-19, SARS-CoV-2, COVID-19 symptoms

## Abstract

Background: Long COVID has become a central public health concern. This study characterized the effectiveness of BNT162b2 BA.4/5 bivalent COVID-19 vaccine (bivalent) against long COVID symptoms. Methods: Symptomatic US adult outpatients testing positive for SARS-CoV-2 were recruited between 2 March and 18 May 2023. Symptoms were assessed longitudinally using a CDC-based symptom questionnaire at Week 4, Month 3, and Month 6 following infection. The odds ratio (OR) of long COVID between vaccination groups was assessed by using mixed-effects logistic models, adjusting for multiple covariates. Results: At Week 4, among 505 participants, 260 (51%) were vaccinated with bivalent and 245 (49%) were unvaccinated. Mean age was 46.3 years, 70.7% were female, 25.1% had ≥1 comorbidity, 43.0% prior infection, 23.0% reported Nirmatrelvir/Ritonavir use. At Month 6, the bivalent cohort had 41% lower risk of long COVID with ≥3 symptoms (OR: 0.59, 95% CI, 0.36–0.96, *p* = 0.034) and 37% lower risk of ≥2 symptoms (OR: 0.63, 95% CI, 0.41–0.96, *p* = 0.030). The bivalent cohort reported fewer and less durable symptoms throughout the six-month follow-up, driven by neurologic and general symptoms, especially fatigue. Conclusions: Compared with unvaccinated participants, participants vaccinated with the bivalent were associated with approximately 40% lower risk of long COVID and less symptom burden over the six-month study duration.

## 1. Background

Long COVID is a multi-system syndrome that could affect individuals following SARS-CoV-2 infection, regardless of age or severity of symptoms experienced during the acute phase of the disease [1]. While there is no single definition, the US Centers for Disease Control and Prevention (CDC) refers to long COVID as a wide range of symptoms and conditions persisting or emerging beyond four weeks following SARS-CoV-2 infection, which are not explained by an alternative diagnosis [1]. However, due to the lack of a universally agreed-upon definition, population estimates can vary substantially between studies based on patient characteristics, COVID-19 variant, and study design and methods [1,2,3,4,5,6].

Long COVID has been associated with significant functional and socioeconomic limitations for patients, as well as substantial public health and economic impact for the healthcare system and society as a whole [7,8,9]. A holistic assessment of the impact of SARS-CoV-2 vaccination should go beyond the acute phase of infection to include quantifying its impact on long COVID. Findings from recent systematic literature reviews and individual studies suggest that vaccination against SARS-CoV-2 may confer protection or amelioration of long COVID symptoms [10,11,12,13,14,15]. For example, Byambasuren, et al. (2023) found that 10 out of 12 studies showed a significant reduction in the incidence of long COVID when patients had been vaccinated before infection with SARS-CoV-2 [10]. However, several of the included studies were conducted in specific populations, such as healthcare workers or veterans, or reported data based on the use of the original monovalent vaccines only [12,13,14].

Updated bivalent formulations of COVID-19 vaccines were authorized in late 2022 to protect against both the original strain and BA.4/5 Omicron sub-lineages, replacing the original monovalent formulations [16]. There are limited data describing the impact of this adapted bivalent formulation on long COVID symptoms. Evidence is needed to understand how these updated formulations impact long COVID symptoms across broad populations, especially as new variants and sub-lineages continue to emerge.

Our initial analyses of a nationwide prospective study showed that among symptomatic outpatient individuals, the BNT162b2 BA.4/5 bivalent COVID-19 vaccine had a protective effect against acute COVID-19 symptoms up to four weeks following infection. [17]. This analysis presents the long-term results of this study, focused on long COVID outcomes through the six months following infection, which were assessed among groups defined by BNT162b2 BA.4/5 bivalent COVID-19 vaccination status.

## 2. Methods

### 2.1. Study Design and Cohorts

A full description of the study design was previously published (clinicaltrials.gov NCT05160636) [17]. This survey-based prospective patient-reported outcomes (PRO) study recruited US adults ≥18 years old with a positive SARS-CoV-2 test via either positive reverse transcription-polymerase chain reaction (RT-PCR) or rapid antigen and self-reporting one or more symptoms associated with acute SARS-CoV-2 infection when testing at a retail pharmacy testing location [17].

Recruitment was carried out between 03/02/2023 and 05/18/2023, during the predominance of the XBB Omicron sub-lineage in the US. Follow-up among those who consented occurred through 11/25/2023. Initial results for the acute phase (defined as up to 4 weeks following infection) were previously published [17]. This analysis presents the long-term results with outcomes until Month 6 post-infection.

As previously described [17], study participants were assigned to two analysis cohorts based on their self-reported pre-infection COVID-19 vaccination history. Participants reporting a vaccination date of 9/1/22 or later for their latest Pfizer-BioNTech COVID-19 vaccine booster were included in the “bivalent” cohort, considering that, since 9/1/22, the only formulation available in the US for COVID-19 vaccines was the adapted bivalent formulation [16]. Participants reporting receipt of any non-BNT162b2 bivalent vaccine were excluded. Participants were included in the “unvaccinated/not-up-to-date” cohort if they either: (1) did not report receipt of any COVID-19 vaccine before testing, or (2) reported having received their last original monovalent dose >12 months before enrollment, considered to be past the time of assumed vaccine-induced immunity [18]. As such, the “Unvaccinated” cohort consisted of both unvaccinated and not up-to-date participants. These two study cohorts are defined as “bivalent” and “unvaccinated”, with these terms used throughout this report.

### 2.2. Baseline Characteristics and Symptoms

Baseline characteristics were gathered via self-reported responses to the CVS Health screening questionnaire administered to individuals scheduling a SARS-CoV-2 test at a CVS site. The characteristics included demographics, underlying comorbidities, COVID-19 vaccination history, work and/or residency in a high-risk or healthcare setting, the Social Vulnerability Index (SVI), antiviral medications use, and symptoms as defined by the CDC’s list of COVID-19 symptoms [19,20].

### 2.3. Long COVID Symptoms

The assessment of long COVID symptoms relied on a questionnaire including 30 symptoms based on the 2022 CDC list [1]. The questionnaire was administered at Week 4, Month 3, and Month 6 after a positive test and enrollment. In alignment with the CDC’s definition, Week 4 was defined as the start of long COVID [1]. The questionnaire assessed the presence of general symptoms (tiredness or fatigue that interferes with daily life, symptoms that become worse after physical or mental activities also known as “post-exertional malaise”, fever, general pain/discomfort, chills or exercise intolerance), respiratory and heart symptoms (difficulty breathing or shortness of breath, cough, chest pain, sore throat, fast-beating or pounding heart also known as heart palpitations), neurologic symptoms (difficulty thinking or concentrating sometimes referred to as “brain fog”, headache, sleep problems, dizziness when you stand up referred to as ‘lightheadedness’, vertigo, pins-and-needles feeling, change in smell or taste, mood changes, memory loss, confusion, depression or anxiety), digestive symptoms (diarrhea, stomach pain, nausea with or without vomiting, loss of appetite), and other symptoms (changes in menstrual cycles, rash, joint or muscle pain, hair loss) (Supplementary Table S1).

The current analysis focused on the long COVID cohort, defined as those individuals that self-reported experiencing ≥1 long COVID symptom at the time of their first long COVID symptoms survey, administered 4 weeks after their initial laboratory-confirmed infection. The long COVID symptoms survey was also subsequently administered at Month 3 and Month 6 following infection. The long COVID analyses assessed the point prevalence of long COVID at Week 4, Month 3, and Month 6 as the occurrence of ≥2 or ≥3 symptoms consistent with long COVID.

### 2.4. Statistical Methods

Baseline characteristics were analyzed with descriptive statistics. Variables were described using means and standard deviations (SDs) (continuous) and frequency and proportions (categorical). Statistical tests to determine differences were used (*t*-tests for continuous variables, chi-square statistics for categorical variables) [21]. Fisher’s exact tests were used for 2-by-2 tables. Freeman–Halton tests for r-by-c tables were used when the expected cell frequency count was less than 5 [22]. All the *p* values were two-sided.

Mixed-effects logistic models were used to examine the impact of vaccination status of long COVID [23]. The candidate model covariates were time, vaccination status and interaction of time by vaccination status, demographic characteristics (age, gender, region, race/ethnicity, social vulnerability), previously tested positive for COVID-19, ≥1 comorbidity, and Nirmatrelvir/Ritonavir prescription received as treatment for the infection they tested positive for at baseline. The assessment time by subject was fitted as a categorical covariate with an unstructured correlation matrix. Odds ratios of long COVID symptoms between the bivalent cohort and unvaccinated cohort were estimated for each assessment time. The same modelling approach was applied to each long COVID symptom or symptom category.

All analyses were conducted with SAS Version 9.4 (SAS Institute, Cary, NC, USA). The study report is aligned to the Strengthening the Reporting of Observational Studies in Epidemiology (STROBE) reporting guideline [24].

## 3. Results

### 3.1. Study Population

At Week 4, 505 study participants completed the first long COVID survey and reported current symptoms of long COVID. Of these, 260 (51.5%) participants were vaccinated with bivalent and 245 (48.5%) were unvaccinated. Baseline characteristics of these participants are presented in Table 1.

The participants were 46.3 years old on average, 70.7% were female, 25.1% reported at least one comorbidity, and 40.4% reported prior infection. Compared with the unvaccinated cohort, the bivalent cohort was older (mean age: 42.4 vs. 50.0 years; *p* < 0.001), had lower social vulnerability (mean SVI: 0.49 vs. 0.39; *p* < 0.001), and had fewer acute COVID-19 symptoms on the index day (mean: 5.7 vs. 5.0; *p* = 0.001). Both cohorts were also significantly different with regard to race/ethnicity (*p* = 0.006). Compared with the unvaccinated cohort, a higher proportion of participants in the bivalent cohort reported having ≥ 1 comorbidity (18.8% vs. 31.2%, *p* = 0.001) and a higher mean number of comorbidities (mean: 0.30 vs. 0.46; *p* = 0.015). Compared with the unvaccinated cohort, a significantly higher proportion of participants in the bivalent cohort were previously prescribed Nirmatrelvir/Ritonavir (17.1% vs. 28.5%; *p* = 0.003). The vaccinated cohort had received their bivalent vaccine on average 165 days prior to study enrollment. The unvaccinated cohort, which comprised a portion of participants not up to date on their COVID-19 vaccination, had received their last vaccine dose, on average, 546 days prior to enrollment. Both cohorts generally looked comparable in terms of sex, previous infection, and levels of risk in the workplace and household settings. Consistent with the study inclusion criteria and the long COVID definition used, all study participants self-reported experiencing ≥1 COVID-19 acute symptoms during the acute phase, and self-reported persistence or development of symptoms consistent with long COVID at long COVID start (Week 4 after initial infection).

### 3.2. Long COVID-19 Risk and Symptoms

#### 3.2.1. Time Trends through Month 6

**Figure 1:**
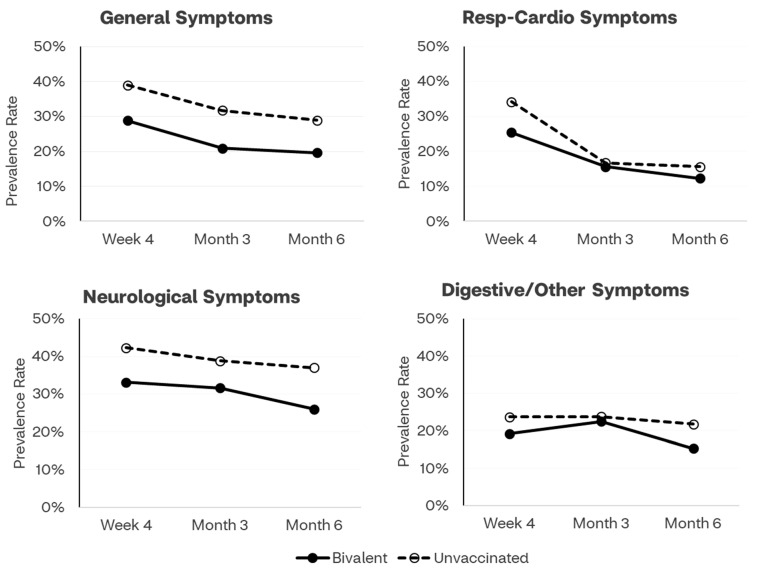
Prevalence of long COVID symptoms by COVID-19 vaccination status and symptoms category across time points (Week 4, Month 3, Month 6). The category “Other” included: rash, joint or muscle pain, hair loss, changes in menstrual cycles.

Time trends of prevalence rates of long COVID symptoms by vaccination status and by category of symptoms are presented in Figure 1. Across all time points, the symptoms prevalence line for the bivalent cohort is below and clearly separated from the line presenting trends for the unvaccinated cohort, for general, neurologic, and digestive/other symptoms.

#### 3.2.2. Week 4

At the start of the long COVID assessment (Week 4) study, participants reported a mean of 2.6 symptoms. The bivalent cohort reported a numerically lower mean number of symptoms (2.4 vs. 2.9, *p* = 0.076). The prevalence of long COVID was significantly lower for the bivalent cohort compared with the unvaccinated cohort (≥3 symptoms: 24.2% vs. 35.5%, *p* = 0.006; ≥2 symptoms: 31.5% vs. 47.8%, *p* = 0.0002). The prevalence of general symptoms was significantly lower for the bivalent cohort compared with the unvaccinated cohort (28.8% vs. 39.0%, *p* = 0.016), as well as the prevalence of respiratory and heart symptoms (25.4% vs. 34.1%, *p* = 0.031) and neurologic symptoms (33.1% vs. 42.3%, *p* = 0.033).

**Figure 2:**
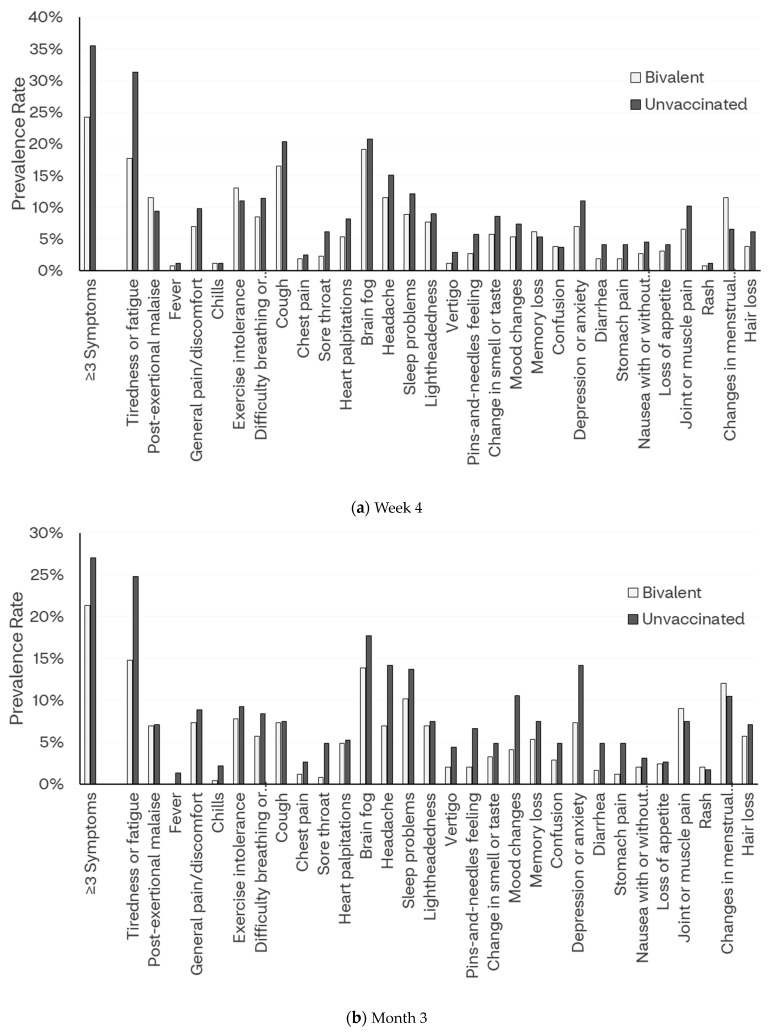

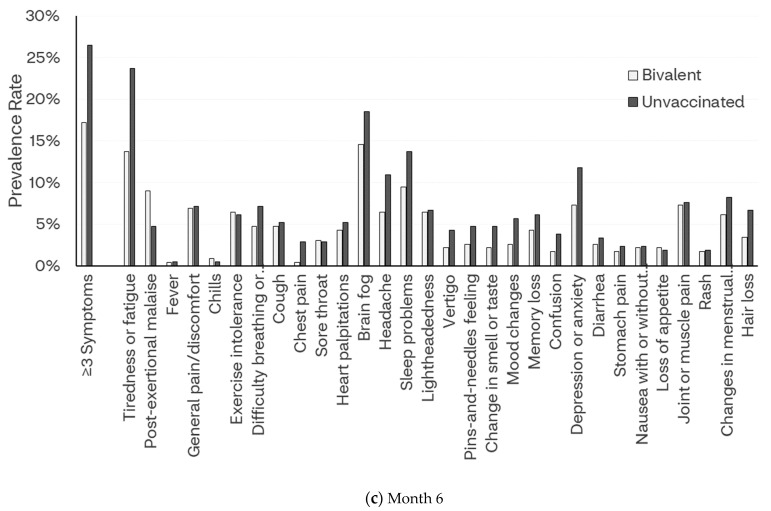
Prevalence of long COVID symptoms by COVID-19 vaccination status.

The proportions of all long COVID symptoms reported at Week 4 were numerically lower in the bivalent cohort, except for symptoms including memory loss, confusion, changes in menstrual cycle, and hair loss. Symptoms of tiredness or fatigue that interfere with daily life (17.7% vs. 31.4%) and sore throat (2.3% vs. 6.1%) were significantly lower for the bivalent cohort compared with the unvaccinated cohort (*p* < 0.05) [Figure 2a and Supplementary Table S1].

#### 3.2.3. Month 3

Overall, the mean number of symptoms declined to 2.4 symptoms at Month 3. On average, the bivalent cohort reported significantly fewer symptoms compared with the unvaccinated cohort (mean: 2.1 vs. 2.8, *p* = 0.028). The prevalence of long COVID was numerically lower, although not statistically significantly different for the bivalent cohort compared with the unvaccinated cohort (≥3 symptoms: 21.3% vs. 27.0%, *p* = 0.150; ≥2 symptoms: 29.1% vs. 36.7%, *p* = 0.078).

Quite similar to Week 4, at Month 3 the proportions of all symptoms were numerically lower in the bivalent cohort, except for rash. Several symptoms were significantly lower for the bivalent cohort compared with the unvaccinated cohort (*p* < 0.05): tiredness or fatigue that interferes with daily life (14.8% vs. 24.8%), sore throat (0.8% vs. 4.9%), headache (7.0% vs. 14.2%), pins-and-needles feeling (2.1% vs. 6.6%), mood changes (4.1% vs. 10.6%), depression or anxiety (7.4% vs. 14.2%), diarrhea (1.6% vs. 4.9%), and stomach pain (1.2% vs. 4.9%) [Figure 2b and Supplementary Table S1].

#### 3.2.4. Month 6

Overall, the mean number of symptoms declined to 2.2 symptoms at Month 6. On average, the bivalent cohort reported a numerically lower, although not statistically significant, mean number of symptoms (2.0 vs. 2.4, *p* = 0.115). The prevalence of long COVID was significantly lower for the bivalent cohort compared with the unvaccinated cohort (≥3 symptoms: 17.2% vs. 26.5%, *p* = 0.017; ≥2 symptoms: 25.8% vs. 37.0%, *p* = 0.011). Directionally, the proportions of all symptoms were numerically lower in the bivalent cohort, except for post-exertional malaise, chills, exercise intolerance, sore throat, and loss of appetite. Symptoms of tiredness or fatigue that interfere with daily life (13.7% vs. 23.7%) and chest pain (0.4% vs. 2.8%) were significantly lower for the bivalent cohort compared with the unvaccinated cohort (*p* < 0.05) [Figure 2c and Supplementary Table S1]. Notably, the symptoms of fatigue/tiredness were consistently lower in the bivalent vaccination group through Month 6 (Supplementary Table S1).

### 3.3. Relationship between Vaccination Status and Risk of Long COVID

Table 2 shows the association between vaccination status and the risk of long COVID six months after the initial infection. The odds ratios (OR) analyses based on observed data showed that, compared to no vaccination, bivalent vaccination was associated with a 41% reduced risk of long COVID expressed as ≥3 symptoms (OR 0.59, 95% CI 0.39–0.89, *p* = 0.011) and with a 43% reduced risk of long COVID expressed as ≥2 symptoms (OR 0.57, 95% CI 0.36–0.91, *p* = 0.017). The logistic regression model (Supplementary Table S2) provided similar results, with the bivalent cohort associated with 41% reduced odds of long COVID expressed as ≥3 symptoms (OR 0.59, 95% CI 0.36–0.96, *p* = 0.034), and 37% reduced odds of long COVID expressed as ≥2 symptoms (OR 0.63, 95% CI 0.41–0.96, *p* = 0.030). These results were driven mainly by general and neurologic symptoms.

### 3.4. Frequency of Symptoms

**Figure 3:**
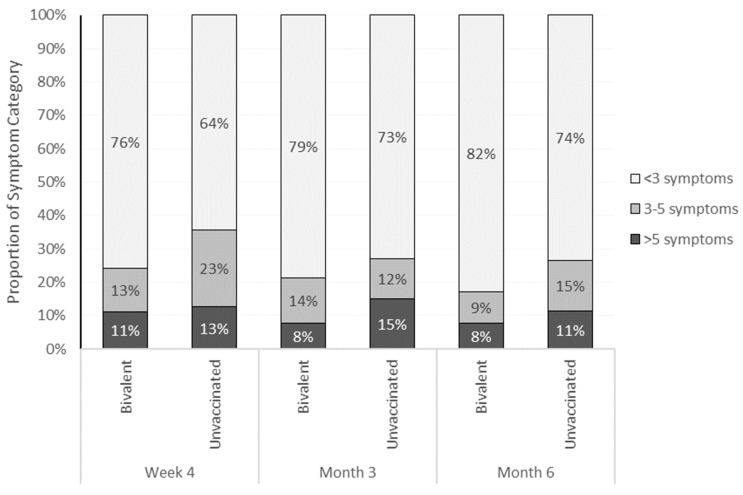
Distribution of study participants across ordinal categories of number of symptoms and COVID-19 vaccination status, across time points (Week 4, Month 3, Month 6). For each vertical bar chart, the sum of the percentages equals 100% (allowing for rounding).

The study participants were stratified in three ordinal categories of self-reported symptoms: <3, 3–5, >5 symptoms. The bivalent cohort presented with lower symptom burden compared with the unvaccinated cohort. This was observed consistently across all time points (Figure 3 and Supplementary Table S1).

## 4. Discussion

This longitudinal prospective survey-based study, conducted among symptomatic adult outpatients with laboratory-confirmed SARS-CoV-2 infections, found that, compared with the unvaccinated group, the cohort of patients that were vaccinated with the BNT162b2 BA.4/5 bivalent COVID-19 vaccine was associated with lower prevalence rates, odds and burden of long COVID. The odds of having at least two or three symptoms consistent with long COVID six months after the initial infection were reduced by approximately 40% by the bivalent vaccine. This effect was mainly driven by general and neurologic symptoms; however, throughout the study period, almost all symptoms were less prevalent or persistent in the bivalent cohort than among the unvaccinated. Further, the number of COVID-19 symptoms was lower among those vaccinated with the bivalent vaccine, across all time points.

To our knowledge, this is the first study that shows the impact of bivalent vaccination on long COVID outcomes. While side-by-side comparisons with other studies are impaired by design and methodological differences, our results are generally in line with prior work on long COVID. Our observed prevalence rates of long COVID are in line with published estimates among non-hospitalized US patients [2,3,4,5,6,7,15,25,26]. Four weeks after the initial infection, approximately 30% of study participants reported experiencing at least three symptoms consistent with long COVID, and 40% reported experiencing at least two symptoms. Those percentages reduced to, respectively, 21% and 33% six months after the initial infection. These results are within ranges found in other studies reporting a prevalence of long COVID from 10% to 50% between one and six months following the initial infection and onset of symptoms. However, while some of those studies included asymptomatic patients [4,25], our study included exclusively the symptomatic population, with 100% of participants reporting ≥1 acute symptom at baseline (per protocol), and ≥1 symptom consistent with long COVID four weeks after infection.

Despite differences in long COVID symptom lists across similar studies, our observed mean symptom count is also in line with published work: the study participants reported a mean of 2.6 symptoms at Week 4 following the initial infection, declining to 2.2 symptoms six months following infection. Other studies reported a mean or median of three to four symptoms [2,3,4,5,6,7,15,26].

The observed differences in long COVID symptoms outcomes by vaccination status support other investigations suggesting that vaccination is associated with reduced risk for long COVID [2,3,4,5,6,7,15,26]. In this study, we found that six months after the initial infection, the bivalent cohort had approximately a 40% lower risk of long COVID, defined as either the presence of at least two or three symptoms. High heterogeneity across studies has precluded meta-analyses in several systematic literature reviews [10,11]. Three meta-analyses reported that patients who had been vaccinated against COVID-19 with at least one or two doses of the original monovalent COVID-19 vaccines had 40–60% reduced risk of developing long COVID compared with patients who were not vaccinated [27,28,29]. In our prior survey-based study assessing the effects of the original monovalent vaccination on the risk of long COVID [18], the model-based results yielded 64% reduced odds of long COVID for those boosted vs. those unvaccinated.

We emphasize that our study is of an observational nature and our findings should be interpreted in the context of several limitations, despite relative consistency with the prior literature. As previously described [17], study limitations included female over-representation, the relatively healthy status of the source population, and the fact that the study included adults only. These characteristics generally aligned with similar survey-based US studies investigating long COVID outcomes. However, while the results may be generalizable to a large part of the adult symptomatic US general population [2,3,4,5,25], they do not generalize to children, inpatient, and high-risk populations. The study groups had different baseline characteristics (age, race, SVI, comorbidities) and antiviral use. We could not sufficiently assess levels of immunity, variations in healthcare access, behavioral differences, and social determinants of health. Despite adjusting for several covariates, including prior infection and SVI, risk of residual confounding may remain. All the data collected were self-reported, and were, therefore, subject to missingness, errors, recall bias, social desirability bias, and selection bias associated with survey attrition. Of the 504 participants, 12% discontinued at Month 6, possibly because of survey burden. While this drop-off rate was relatively consistent with similar survey-based studies [5,26], this study did not employ validated methods to assess self-report bias. As such, bias could not be controlled for nor adjusted in the analyses, potentially affecting data accuracy and the interpretation of data variance. While long COVID research is benefiting from studies that, like ours, rely on self-reported symptoms and vaccination data as the primary source of data [2,3,4,5,25], understanding sources of bias accompanying self-reported data is key to limit their implications.

Further, the study did not explore differences between groups in terms of digital health literacy. The study findings may not be applicable to prior or future variants or sub-lineages. Lastly, our study is limited by the lack of a universal definition of long COVID. The primary analyses of survey-based studies have generally assessed the prevalence of long COVID based on the presence of one or more COVID-19 symptoms beyond the initial month of infection [2,3,4,5,6]. While our analyses also used long COVID symptom presence for inclusivity, future sensitivity analyses and research should differentiate the assessment of new-onset symptoms vs. persistent or waxing and waning symptoms and explore subgroup analyses based on factors modifying vaccine effectiveness, including potential synergistic effects with antiviral medications. Future studies should also evaluate pre-existing symptoms to limit the risk that those could be erroneously attributed to SARS-CoV-2 infection, and to enable more precise medical assessments. Moreover, future research avenues could explore additional health outcomes such as the assessment of severity of symptoms and time to symptom resolution. Lastly, future studies could use a longer follow-up period beyond six months to more comprehensively capture the burden of long COVID and generate additional insights into long-term vaccine effectiveness.

The BNT162b2 BA.4/5 bivalent COVID-19 vaccine has been shown to be effective at preventing a range of COVID-19 infection outcomes in real-world data [30]. These data support the potential protective association of BNT162b2 BA.4/5 bivalent vaccination in reducing long COVID sequela. As the COVID-19 epidemic continues, concerns about long-term health impacts and ways to prevent and treat long COVID persist [7]. As such, these data highlight the importance of COVID-19 prevention and treatment, including staying up to date with recommended COVID-19 vaccinations, and can help support long COVID care efforts.

## 5. Conclusions

This study showed that long COVID symptoms persisted for months after the initial infection among symptomatic adults. Receipt of the BNT162b2 BA.4/5 bivalent vaccine was associated with a diminished risk and burden of long COVID symptoms. The study findings were broadly consistent with the existing literature and provided novel insights into the clinical effectiveness of the bivalent vaccine against disease symptoms experienced after a breakthrough infection. These results reinforce the importance of staying up to date with recommended COVID-19 vaccinations and support long COVID prevention and management efforts.

## Figures and Tables

**Table 1:** Baseline Demographic and Clinical Characteristics of Study Participants at Week 4.

	All	BNT162b2	Unvaccinated	*p* ^a^
Total, n (%)	505	260	245	
Age, years				
Mean, SD	46.3 (15.5)	50.0 (16.0)	42.4 (14.0)	<0.001
18–29	76 (15.0%)	28 (10.8%)	48 (19.6%)	<0.001
30–49	220 (43.6%)	99 (38.1%)	121 (49.4%)	
50–64	125 (24.8%)	69 (26.5%)	56 (22.9%)	
≥65	84 (16.7%)	64 (24.6%)	20 (8.2%)	
Gender				0.086
Female	357 (70.7%)	173 (66.5%)	184 (75.1%)	
Male	144 (28.5%)	84 (32.3%)	60 (24.5%)	
Unknown	4 (0.8%)	3 (1.2%)	1 (0.4%)	
Race/Ethnicity				0.006
White or Caucasian	305 (60.4%)	168 (64.6%)	137 (55.9%)	
Black or African American	40 (7.9%)	15 (5.8%)	25 (10.2%)	
Hispanic	73 (14.5%)	26 (10.0%)	47 (19.2%)	
Asian	49 (9.7%)	31 (11.9%)	18 (7.4%)	
Other	38 (7.5%)	20 (7.7%)	18 (7.4%)	
US Geographic Region				0.053
Northeast	68 (13.5%)	38 (14.6%)	30 (12.2%)	
South	201 (39.8%)	89 (34.2%)	112 (45.7%)	
Midwest	113 (22.4%)	67 (25.8%)	46 (18.8%)	
West	123 (24.4%)	66 (25.4%)	57 (23.3%)	
Social vulnerability index, Mean (SD) ^b^	0.44 (0.23)	0.39 (0.22)	0.49 (0.23)	<0.001
Previously tested positive	204 (40.4%)	98 (37.7%)	106 (43.3%)	0.202
Live or work in high-risk setting	25 (5.0%)	10 (3.9%)	15 (6.1%)	0.239
Work in healthcare	75 (14.9%)	33 (12.7%)	42 (17.1%)	0.160
Self-reported comorbidity				
Number of comorbidities, Mean (SD)	0.38 (0.75)	0.46 (0.79)	0.30 (0.70)	0.015
Asthma or chronic lung disease	25 (5.0%)	17 (6.5%)	8 (3.3%)	0.090
Immunocompromised conditions or weakened immune system ^c^	2 (0.4%)	1 (0.4%)	1 (0.4%)	0.966
Diabetes	27 (5.4%)	17 (6.5%)	10 (4.1%)	0.220
Heart conditions or hypertension	79 (15.6%)	49 (18.8%)	30 (12.2%)	0.041
Overweight or obesity	60 (11.9%)	36 (13.8%)	24 (9.8%)	0.160
At least 1 comorbidity	127 (25.1%)	81 (31.2%)	46 (18.8%)	0.001
Mean days since last vaccine dose, SD	337 (209)	165 (46)	546 (121)	<0.001
Nirmatrelvir/Ritonavir use, n (%)	116 (23.0%)	74 (28.5%)	42 (17.1%)	0.003
Acute COVID-19 symptoms on index day ^d^	505 (100%)	260 (100%)	245 (100%)	
Mean number of symptoms, SD	5.3 (2.3)	5.0 (2.3)	5.7 (2.3)	0.001

SD: Standard Deviation; CMS: Centers for Medicare and Medicaid Services; IQR: Interquartile Range.^a^ *p* value for the comparison between BNT162b2 and Unvaccinated. ^b^ SVI is a score that ranges from 0 to 1. Higher values correspond to higher vulnerability. ^c^ Immunocompromised conditions comprise conditions that result in a weakened immune system, including kidney failure or end stage renal disease, and indicators of compromised immune system (such as from the use of immune-compromising drugs, solid organ or blood stem cell transplant, HIV, or other conditions). ^d^ The index day is the day of receipt of the COVID-19 test nasal swab test.

**Table 2:** Long COVID Symptoms at Month 6 by COVID-19 Vaccination Status: observed and adjusted model-based results ^a^.

	All (n = 444)	BNT162b2 (n = 233)	Unvaccinated (n = 211)	Observed		Model-Based	
				OR (95% CI)	*p* Value	OR (95% CI)	*p* Value
Long COVID							
≥2 symptoms	138 (31.1%)	60 (25.8%)	78 (37.0%)	0.59 (0.39, 0.89)	0.011	0.63 (0.41, 0.96)	0.030
≥3 symptoms	96 (21.6%)	40 (17.2%)	56 (26.5%)	0.57 (0.36, 0.91)	0.017	0.59 (0.36, 0.96)	0.034
Symptom category							
General symptoms	107 (24.0%)	46 (19.6%)	61 (28.9%)	0.60 (0.39, 0.94)	0.021	0.63 (0.40, 0.99)	0.048
Respiratory and cardio symptoms	62 (13.9%)	29 (12.3%)	33 (15.6%)	0.77 (0.45, 1.31)	0.315	0.80 (0.47, 1.38)	0.424
Neurologic symptoms	139 (31.2%)	61 (26.0%)	78 (37.0%)	0.60 (0.40, 0.91)	0.012	0.60 (0.39, 0.92)	0.018
Digestive/other symptoms	82 (18.4%)	36 (15.3%)	46 (21.8%)	0.66 (0.40, 1.06)	0.078	0.69 (0.42, 1.13)	0.137

^a^ Based on mixed-effects logistic models with unstructured correlation matrix. Covariates were sociodemographic characteristics (age, sex, regions, social vulnerability, race/ethnicity), variables for time, vaccination status and interaction of time by vaccination status, as well as ≥1 comorbidity, previously tested positive for COVID-19, and Nirmatrelvir/Ritonavir prescription. OR = odds ratio; CI = confidence interval.

## Data Availability

Aggregated data that support the findings of this study are available upon reasonable request from the corresponding author MDF, subject to review. These data are not publicly available due to them containing information that could compromise research participant privacy/consent.

## Supplementary Materials

The following supporting information can be downloaded at: https://www.mdpi.com/article/10.3390/vaccines12020183/s1, Table S1: Trajectory of Long COVID Symptoms at 1-, 3- and 6-month follow-up; Table S2: Multivariate Logistic Models for Long COVID.

## Abbreviations

CDC	Centers for Disease Control and Prevention;
CI	confidence interval;
SD	standard deviation;
SVI	social vulnerability index.
