# Supplementary material for: Effectiveness of BNT162b2 BA.4/5 Bivalent COVID-19 Vaccine against Long COVID Symptoms: A US Nationwide Study
Source: Vaccines (Basel). 2024 Feb 11;12(2):183. doi: 10.3390/vaccines12020183 (PMC10893330; doi:10.3390/vaccines12020183)
Supplement: Supplementary file 1 [file vaccines-12-00183-s001.zip › vaccines-2844547-supplementary.pdf]

Effectiveness of BNT162b2 BA.4/5 bivalent COVID-19 vaccine against long COVID symptoms: a US nationwide study

Supplemental Material

Supplemental Table S1. Trajectory of Long COVID Symptoms at 1-, 3- and 6-month follow-up

	All	BNT162b2	Unvaccinated	P [a]	P [b]
**Week 4**					
n	505	260	245		
Mean (SD)	2.6 (3.1)	2.4 (3.0)	2.9 (3.2)	0.076	
≥3 symptoms	150 (29.7%)	63 (24.2%)	87 (35.5%)	0.006	0.015
General symptoms					
≥1 symptom	171 (33.8%)	75 (28.8%)	96 (39.0%)	0.016	0.069
Tiredness or fatigue that interferes with daily life	123 (24.4%)	46 (17.7%)	77 (31.4%)	0.000	0.002
Symptoms that get worse after physical or mental activities (also known as “post-exertional malaise”)	53 (10.5%)	30 (11.5%)	23 (9.4%)	0.431	0.335
Fever	5 (1.0%)	2 (0.8%)	3 (1.2%)	0.606	-
General pain/discomfort	42 (8.3%)	18 (6.9%)	24 (9.8%)	0.243	0.313
Chills	6 (1.2%)	3 (1.2%)	3 (1.2%)	0.942	0.960
Exercise intolerance	61 (12.1%)	34 (13.1%)	27 (11.0%)	0.479	0.285
Respiratory and cardiac					
≥1 symptom	150 (29.6%)	66 (25.4%)	84 (34.1%)	0.031	0.059
Difficulty breathing or shortness of breath	50 (9.9%)	22 (8.5%)	28 (11.4%)	0.265	0.488
Cough	93 (18.4%)	43 (16.5%)	50 (20.4%)	0.262	0.133
Chest pain	11 (2.2%)	5 (1.9%)	6 (2.5%)	0.686	0.697
Sore throat	21 (4.2%)	6 (2.3%)	15 (6.1%)	0.032	0.025
Fast-beating or pounding heart (also known as heart palpitations)	34 (6.7%)	14 (5.4%)	20 (8.2%)	0.213	0.375
Neurologic					
≥1 symptom	190 (37.5%)	86 (33.1%)	104 (42.3%)	0.033	0.035
Difficulty thinking or concentrating (sometimes referred to as “brain fog”)	101 (20.0%)	50 (19.2%)	51 (20.8%)	0.656	0.944
Headache	67 (13.3%)	30 (11.5%)	37 (15.1%)	0.238	0.309
Sleep problems	53 (10.5%)	23 (8.9%)	30 (12.2%)	0.213	0.228
Dizziness when you stand up (lightheadedness)	42 (8.3%)	20 (7.7%)	22 (9.0%)	0.601	0.597
Vertigo	10 (2.0%)	3 (1.2%)	7 (2.9%)	0.170	0.189
Pins-and-needles feeling	21 (4.2%)	7 (2.7%)	14 (5.7%)	0.089	0.155
Change in smell or taste	36 (7.1%)	15 (5.8%)	21 (8.6%)	0.221	0.081

Mood changes	32 (6.3%)	14 (5.4%)	18 (7.4%)	0.366	0.696
Memory loss	29 (5.7%)	16 (6.2%)	13 (5.3%)	0.682	0.486
Confusion	19 (3.8%)	10 (3.9%)	9 (3.7%)	0.919	0.780
Depression or anxiety	45 (8.9%)	18 (6.9%)	27 (11.0%)	0.106	0.251
Digestive and Other					
≥1 symptom	108 (21.4%)	50 (19.2%)	58 (23.7%)	0.224	0.445
Diarrhea	15 (3.0%)	5 (1.9%)	10 (4.1%)	0.153	0.388
Stomach pain	15 (3.0%)	5 (1.9%)	10 (4.1%)	0.153	0.434
Nausea with or without vomiting	18 (3.6%)	7 (2.7%)	11 (4.5%)	0.276	0.286
Loss of appetite	18 (3.6%)	8 (3.1%)	10 (4.1%)	0.543	0.556
Joint or muscle pain	42 (8.3%)	17 (6.5%)	25 (10.2%)	0.136	0.057
Rash	5 (1.0%)	2 (0.8%)	3 (1.2%)	0.606	0.253
Changes in menstrual cycles	22 (8.6%)	12 (11.5%)	10 (6.6%)	0.164	0.172
Hair loss	25 (5.0%)	10 (3.9%)	15 (6.1%)	0.239	0.489
**Month 3**					
n	470	244	226		
Mean (SD)	2.4 (3.1)	2.1 (2.4)	2.8 (3.7)	0.028	
≥3 symptoms	113 (24.0%)	52 (21.3%)	61 (27.0%)	0.150	0.318
General symptoms					
≥1 symptom	123 (26.1%)	51 (20.9%)	72 (31.7%)	0.008	0.036
Tiredness or fatigue that interferes with daily life	92 (19.6%)	36 (14.8%)	56 (24.8%)	0.006	0.016
Symptoms that get worse after physical or mental activities (also known as "post-exertional malaise")	33 (7.0%)	17 (7.0%)	16 (7.1%)	0.962	0.910
Fever	3 (0.6%)	0 (0.0%)	3 (1.3%)	0.071	-
General pain/discomfort	38 (8.1%)	18 (7.4%)	20 (8.9%)	0.559	0.725
Chills	6 (1.3%)	1 (0.4%)	5 (2.2%)	0.082	0.191
Exercise intolerance	40 (8.5%)	19 (7.8%)	21 (9.3%)	0.559	0.935
Respiratory and cardiac					
≥1 symptom	76 (16.1%)	38 (15.6%)	38 (16.7%)	0.731	0.990
Difficulty breathing or shortness of breath	33 (7.0%)	14 (5.7%)	19 (8.4%)	0.258	0.569
Cough	35 (7.5%)	18 (7.4%)	17 (7.5%)	0.952	0.764
Chest pain	9 (1.9%)	3 (1.2%)	6 (2.7%)	0.260	0.276
Sore throat	13 (2.8%)	2 (0.8%)	11 (4.9%)	0.008	0.029
Fast-beating or pounding heart (also known as heart palpitations)	24 (5.1%)	12 (4.9%)	12 (5.3%)	0.847	0.864
Neurologic					
≥1 symptom	165 (35.0%)	77 (31.6%)	88 (38.8%)	0.101	0.167
Difficulty thinking or concentrating (sometimes referred to as "brain fog")	74 (15.7%)	34 (13.9%)	40 (17.7%)	0.263	0.511
Headache	49 (10.4%)	17 (7.0%)	32 (14.2%)	0.011	0.051

Sleep problems	56 (11.9%)	25 (10.2%)	31 (13.7%)	0.246	0.300
Dizziness when you stand up (lightheadedness)	34 (7.2%)	17 (7.0%)	17 (7.5%)	0.817	0.870
Vertigo	15 (3.2%)	5 (2.1%)	10 (4.4%)	0.143	0.215
Pins-and-needles feeling	20 (4.3%)	5 (2.1%)	15 (6.6%)	0.014	0.050
Change in smell or taste	19 (4.0%)	8 (3.3%)	11 (4.9%)	0.382	0.267
Mood changes	34 (7.2%)	10 (4.1%)	24 (10.6%)	0.006	0.024
Memory loss	30 (6.4%)	13 (5.3%)	17 (7.5%)	0.331	0.665
Confusion	18 (3.8%)	7 (2.9%)	11 (4.9%)	0.259	0.344
Depression or anxiety	50 (10.6%)	18 (7.4%)	32 (14.2%)	0.017	0.079
Digestive and Other					
≥1 symptom	109 (23.1%)	55 (22.5%)	54 (23.8%)	0.748	0.907
Diarrhea	15 (3.2%)	4 (1.6%)	11 (4.9%)	0.047	0.184
Stomach pain	14 (3.0%)	3 (1.2%)	11 (4.9%)	0.021	0.047
Nausea with or without vomiting	12 (2.6%)	5 (2.1%)	7 (3.1%)	0.472	0.528
Loss of appetite	12 (2.6%)	6 (2.5%)	6 (2.7%)	0.893	0.917
Joint or muscle pain	39 (8.3%)	22 (9.0%)	17 (7.5%)	0.557	0.757
Rash	9 (1.9%)	5 (2.1%)	4 (1.8%)	0.825	0.957
Changes in menstrual cycles	27 (11.1%)	12 (12.0%)	15 (10.5%)	0.712	0.758
Hair loss	30 (6.4%)	14 (5.7%)	16 (7.1%)	0.552	0.983
**Month 6**					
n	444	233	211		
Mean (SD)	2.2 (2.6)	2.0 (2.5)	2.4 (2.7)	0.115	
≥3 symptoms	96 (21.6%)	40 (17.2%)	56 (26.5%)	0.017	0.034
General symptoms					
≥1 symptom	107 (24.0%)	46 (19.6%)	61 (28.9%)	0.021	0.048
Tiredness or fatigue that interferes with daily life	82 (18.5%)	32 (13.7%)	50 (23.7%)	0.007	0.013
Symptoms that get worse after physical or mental activities (also known as “post-exertional malaise”)	31 (7.0%)	21 (9.0%)	10 (4.7%)	0.078	0.080
Fever	2 (0.5%)	1 (0.4%)	1 (0.5%)	0.944	-
General pain/discomfort	31 (7.0%)	16 (6.9%)	15 (7.1%)	0.920	0.958
Chills	3 (0.7%)	2 (0.9%)	1 (0.5%)	0.621	0.497
Exercise intolerance	28 (6.3%)	15 (6.4%)	13 (6.2%)	0.905	0.700
Respiratory and cardiac					
≥1 symptom	62 (13.9%)	29 (12.3%)	33 (15.6%)	0.315	0.424
Difficulty breathing or shortness of breath	26 (5.9%)	11 (4.7%)	15 (7.1%)	0.285	0.625
Cough	22 (5.0%)	11 (4.7%)	11 (5.2%)	0.811	0.579
Chest pain	7 (1.6%)	1 (0.4%)	6 (2.8%)	0.041	0.078
Sore throat	13 (2.9%)	7 (3.0%)	6 (2.8%)	0.920	0.984

Fast-beating or pounding heart (also known as heart palpitations)	21 (4.7%)	10 (4.3%)	11 (5.2%)	0.648	0.885
Neurologic					
≥1 symptom	139 (31.2%)	61 (26.0%)	78 (37.0%)	0.012	0.018
Difficulty thinking or concentrating (sometimes referred to as “brain fog”)	73 (16.4%)	34 (14.6%)	39 (18.5%)	0.269	0.426
Headache	38 (8.6%)	15 (6.4%)	23 (10.9%)	0.093	0.137
Sleep problems	51 (11.5%)	22 (9.4%)	29 (13.7%)	0.156	0.168
Dizziness when you stand up (lightheadedness)	29 (6.5%)	15 (6.4%)	14 (6.6%)	0.933	0.860
Vertigo	14 (3.2%)	5 (2.2%)	9 (4.3%)	0.202	0.223
Pins-and-needles feeling	16 (3.6%)	6 (2.6%)	10 (4.7%)	0.222	0.241
Change in smell or taste	15 (3.4%)	5 (2.2%)	10 (4.7%)	0.131	0.109
Mood changes	18 (4.1%)	6 (2.6%)	12 (5.7%)	0.097	0.140
Memory loss	23 (5.2%)	10 (4.3%)	13 (6.2%)	0.375	0.721
Confusion	12 (2.7%)	4 (1.7%)	8 (3.8%)	0.178	0.288
Depression or anxiety	42 (9.5%)	17 (7.3%)	25 (11.8%)	0.102	0.249
Digestive and Other					
≥1 symptom	82 (18.4%)	36 (15.3%)	46 (21.8%)	0.078	0.137
Diarrhea	13 (2.9%)	6 (2.6%)	7 (3.3%)	0.643	0.866
Stomach pain	9 (2.0%)	4 (1.7%)	5 (2.4%)	0.626	0.955
Nausea with or without vomiting	10 (2.3%)	5 (2.2%)	5 (2.4%)	0.874	0.728
Loss of appetite	9 (2.0%)	5 (2.2%)	4 (1.9%)	0.852	0.759
Joint or muscle pain	33 (7.4%)	17 (7.3%)	16 (7.6%)	0.908	0.699
Rash	8 (1.8%)	4 (1.7%)	4 (1.9%)	0.887	0.737
Changes in menstrual cycles	17 (7.3%)	6 (6.1%)	11 (8.2%)	0.547	0.552
Hair loss	22 (5.0%)	8 (3.4%)	14 (6.6%)	0.121	0.250

[a] *P* values of t-test for number of symptoms, Chi-square tests or Fisher’s exact tests when any one cell has an expected frequency less than 5 for individual symptoms and number of symptom category comparing the BNT162b2 cohort and the unvaccinated cohort
[b] Model-based *P* value
SD=Standard Deviation

Supplemental Table S2. Multivariable Logistic Models for Long COVID [a]

Patient Characteristics	Estimate	Standard Error	P value
Intercept	-1.27	0.34	<0.001
Vaccination Status			
Bivalent	-0.51	0.21	0.015
Unvaccinated	Reference		
Assessment Time			
Week 4	Reference		
Month 3	-0.45	0.15	0.004
Month 6	-0.43	0.16	0.008
Vaccination Status by Assessment Time			
Bivalent*Month 3	0.28	0.22	0.203
Bivalent*Month 6	-0.01	0.24	0.955
Age group, years			
18-29	Reference		
30-49	0.65	0.28	0.022
50-64	0.96	0.31	0.002
≥65	0.53	0.35	0.136
Gender			
Male	-0.65	0.21	0.003
Female	Reference		
Social Vulnerability Index category			
<0.25	Reference		
≥0.25 and <0.5	0.28	0.23	0.225
≥0.5 and <0.75	-0.04	0.27	0.895
≥0.75	0.76	0.29	0.009

[a] Long COVID was defined as self-reporting 3 or more long-COVID symptoms at the time of assessment.
